# Whole Genome Sequencing of Staphylococci Isolated From Bovine Milk Samples

**DOI:** 10.3389/fmicb.2021.715851

**Published:** 2021-12-20

**Authors:** Marte Ekeland Fergestad, Fabrice Touzain, Sarne De Vliegher, Anneleen De Visscher, Damien Thiry, Cyrille Ngassam Tchamba, Jacques G. Mainil, Trine L’Abee-Lund, Yannick Blanchard, Yngvild Wasteson

**Affiliations:** ^1^Department of Paraclinical Sciences, Faculty of Veterinary Medicine, Norwegian University of Life Sciences, Oslo, Norway; ^2^Anses, Ploufragan-Plouzané-Niort Laboratory, Unit of Viral Genetics and Biosafety, Ploufragan, France; ^3^M-team and Mastitis and Milk Quality Research Unit, Department of Reproduction, Obstetrics, and Herd Health, Faculty of Veterinary Medicine, Ghent University, Ghent, Belgium; ^4^Bacteriology, Department of Infection and Parasitic Diseases, Faculty of Veterinary Medicine, FARAH Research Centre, Liège University, Liège, Belgium

**Keywords:** non-*aureus* staphylococci, *Staphylococcus aureus*, bovine, whole genome sequencing, antimicrobial resistance (AMR) genes, virulence genes

## Abstract

Staphylococci are among the commonly isolated bacteria from intramammary infections in bovines, where *Staphylococcus aureus* is the most studied species. This species carries a variety of virulence genes, contributing to bacterial survival and spread. Less is known about non-*aureus* staphylococci (NAS) and their range of virulence genes and mechanisms, but they are the most frequently isolated bacteria from bovine milk. Staphylococci can also carry a range of antimicrobial resistance genes, complicating treatment of the infections they cause. We used Illumina sequencing to whole genome sequence 93 staphylococcal isolates selected from a collection of staphylococcal isolates; 45 *S. aureus* isolates and 48 NAS isolates from 16 different species, determining their content of antimicrobial resistance genes and virulence genes. Antimicrobial resistance genes were frequently observed in the NAS species as a group compared to *S. aureus*. However, the lincosamide resistance gene *lnuA* and penicillin resistance gene *blaZ* were frequently identified in NAS, as well as a small number of *S. aureus*. The *erm* genes conferring macrolide resistance were also identified in several NAS isolates and in a small number of *S. aureus* isolates. In most *S. aureus* isolates, no antimicrobial resistance genes were detected, but in five *S. aureus* isolates three to six resistance genes were identified and all five of these carried the *mecA* gene. Virulence genes were more frequently identified in *S. aureus*, which contained on average five times more virulence genes compared to NAS. Among the NAS species there were also differences in content of virulence genes, such as *S. chromogenes* with a higher average number of virulence genes. By determining the content of a large selection of virulence genes and antimicrobial resistance genes in *S. aureus* and 16 different NAS species our results contribute with knowledge regarding the genetic basis for virulence and antimicrobial resistance in bovine staphylococci, especially the less studied NAS. The results can create a broader basis for further research into the virulence mechanisms of this important group of bacteria in bovine intramammary infections.

## Introduction

The genus *Staphylococcus* includes a range of different species (De Buck et al., 2021) some of which are among the most commonly isolated bacteria causing intramammary infections in bovines (Pitkälä et al., 2004; Reksen et al., 2006; Olde Riekerink et al., 2008). For a long time, *Staphylococcus aureus* has been the most recognized staphylococcal species causing both clinical and subclinical mastitis (Østerås et al., 2006; Olde Riekerink et al., 2008). The species is associated with a wide range of genes encoding a large diversity of virulence factors involved in adhesion, host immune evasion and biofilm formation (Foster et al., 2014; Geoghegan and Foster, 2017), toxins promoting inflammation and leukocyte death and exoenzymes cleaving and disabling immune molecules. All these factors contribute to bacterial survival, -spread and nutrient acquisition (Tam and Torres, 2019). However, in recent years, non-*aureus* staphylococci (NAS) have emerged as the most frequently isolated bacterial group from bovine milk in many countries and they are increasingly associated with intramammary infections (Pyörälä and Taponen, 2009; De Vliegher et al., 2012; Condas et al., 2017; De Buck et al., 2021).

Emergence of antimicrobial resistant staphylococci is of growing concern in the dairy industry. These bacteria can carry a large number of resistance determinants, which are often located on mobile genetic elements that facilitates horizontal spread of genes (Malachowa and DeLeo, 2010; Kadlec et al., 2012). NAS are regarded as a potential reservoir for antimicrobial resistance genes that can be transferred to and utilized by *S. aureus* (Otto, 2013; Vitali et al., 2014). Many NAS species are found as commensals on teat apices, hair, nares, vagina, teat, and udder skin and inguinal skin, as well as in the environment in the barn (Taponen et al., 2008; Piessens et al., 2011; De Visscher et al., 2014), potentiating possible interactions with a variety of different bacteria present in the host. Due to these commensal properties, NAS may become exposed to several antimicrobials, not only by agents used for battling staphylococcal infections, but also by agents used to combat other pathogens (Kadlec et al., 2012). Several studies have pointed to some NAS species having a relatively greater impact on udder health, especially *S. chromogenes, S. simulans* and *S. xylosus* (Supré et al., 2011; Fry et al., 2014; Valckenier et al., 2019). However, contrary to *S. aureus*, the virulence factors of NAS, and mechanisms behind NAS’ ability to colonize and infect the bovine udder are poorly described (Pyörälä and Taponen, 2009; Vanderhaeghen et al., 2015; Taponen et al., 2017). The extensive virulence gene profiling of 441 NAS isolates published by Naushad et al. (2019), the comparative study of 24 bovine-associated staphylococci of Åvall-Jääskeläinen et al. (2018) and the study on bovine NAS by Wuytack et al. (2020) are the most important published studies that have explored the virulence factors and virulence mechanisms in bovine NAS species, which warrants further investigation of other NAS collections.

The aim of this study was therefore to describe the genetic background for antimicrobial resistance and virulence in staphylococci from dairy cows. The objectives were to (i) whole genome sequence a selection of NAS and *S. aureus* isolated from bovine milk samples, (ii) determine the isolates’ content of antimicrobial resistance and virulence genes, and (iii) compare the types and variety of virulence genes between NAS species and *S. aureus*.

## Materials and Methods

### Collection and Selection of Isolates

**FIGURE 1:**
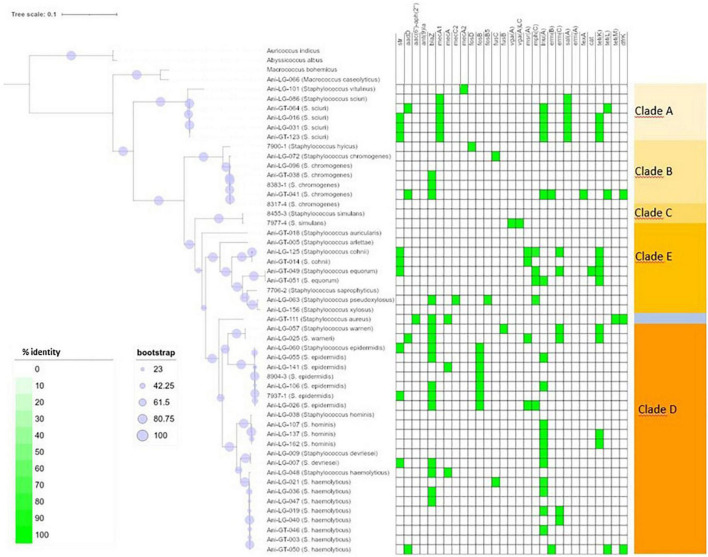
Phylogeny and antimicrobial resistance genes of non-*aureus* staphylococci. Presence of antimicrobial resistance gene is indicated with green square with percent identity indicated by color shades according to the scale.

The isolates originated from a previous study (Fergestad et al., 2021) of bovine staphylococci from Belgian and Norwegian dairy farms, in which 464 lactating cows were sampled from 13 farms in Belgium, and 100 cows with clinical mastitis were sampled from 100 farms in Norway (one cow from each farm). As described in Fergestad et al. (2021), the sampling strategy for that study was adapted to national structures for dairy production and thus varied between countries and regions, but also ensured a diversity of the material. A total of 272 staphylococcal isolates were characterized with regard to their phenotypic antimicrobial resistance, and carriage of the methicillin resistance genes *mecA* and *mecC* and some selected virulence genes were determined by PCR. From this collection, a total of 95 isolates were selected for whole genome sequencing (WGS) according to the following criteria: (i) all *S. aureus* isolates, (ii) representatives of all NAS species, (iii) all isolates positive in PCR for *mecA* or *mecC*. Selection of NAS isolates was further based on phenotypic resistance patterns observed in species with more than one isolate, ensuring that both highly resistant and less resistant isolates were represented. Lastly, it was aimed to achieve an even distribution of isolates originating from the different geographical regions (Norway and Belgium). Altogether, the isolates selected for WGS were 45 *S. aureus* isolates and 50 NAS isolates [identified by Maldi Tof-MS; (Cameron et al., 2017, 2018)] from 16 different species: *S. arlettae*, *S. auricularis*, *S. chromogenes*, *S. cohnii*, *S. devriesei*, *S. epidermidis*, *S. equorum, S. haemolyticus*, *S. hominis*, *S. hyicus*, *S. saprophyticus*, *S. sciuri*, *S. simulans*, *S. vitulinus, S. warneri*, and *S. xylosus*. Background information about the selected isolates is presented in Supplementary Table 1. Some reclassification within the genus *Staphylococcus* has recently been suggested; the reassignment of *S. sciuri* and *S. vitulinus* to a novel genus *Mammaliicoccus* (Madhaiyan et al., 2020) and the novel species *Staphylococcus pseudoxylosus*, closely related to *S. xylosus* (MacFadyen et al., 2019). For the sake of clarity, the suggested *Mammaliicoccus* species are considered as members of genus *Staphylococcus* and *S. pseudoxylosus* is included with the *S. xylosus* in this manuscript. The isolate identified as *S. pseudoxylosus* is, however, shown in the phylogenetic tree of NAS (Figure 1), to illustrate its phylogenetic placement.

### DNA Extraction, Whole Genome Sequencing and Assembly

DNA was extracted using Masterpure*™* Gram Positive DNA Purification Kit (Lucigen, Middleton, WI, United States). Quality and DNA concentrations were determined with Nanodrop 1000 (Thermo Fisher Scientific).

The genomic DNA libraries were prepared for Illumina sequencing according to the manufacturer’s instructions using the Nextera XT kit and sequenced by the NovaSeq 6000 Sequencing System (Illumina, San Diego, CA, United States). The raw read sequences were assembled into contigs with the pipeline Shovill 1.0.4 (Seemann et al., 2020) including trimmomatic 0.38 (Bolger et al., 2014) for the cleaning and annotated using Prokka 1.13.3 (Seemann, 2014).

For LG-048 and LG-101 strains, Nanopore MinION long-read sequencing was performed using the Rapid Barcoding Sequencing kit (Oxford Nanopore) for library preparation. After guppy_gpu base calling, assembly of nanopore reads was performed using Canu 1.8 (Koren et al., 2017). The Illumina reads were cleaned with trimmomatic 0.36 (ILLUMINACLIP:illumina_oligos_and_revcomp:2:30:5:1:true LEADING:3 TRAILING:3 MAXINFO:40:0.2 MINLEN:36 options) and aligned with contigs provided by Canu assembly using BWA 0.7.15-r1140 (arXiv:1303.3997). Pilon 1.23 (Walker et al., 2014) was run on this alignment for preliminary corrections. The final result was the corrected consensus provided by Pilon.

The statistics of assemblies are provided in the Supplementary Table 2.

### Phylogenetic Trees

For creation of alignment and phylogenetic trees of the staphylococcal isolates we included some additional strains of different genera as outgroups to clarify where in the phylogenetic landscape the staphylococcal species were located. These strains were *Macrococcus bohemicus* (NZ_CM009972.1 to NZ_CM009973.1 and NZ_PZJG01000001.1 to NZ_PZJG01000029.1), *Abyssicoccus albus* (NZ_RKRK01000001.1 to NZ_RKRK01000010.1), and *Auricoccus indicus* (NZ_CP019573.1). We ran Panaroo 1.2.3 to analyze core genome to get common genes between the 95 strains (options “–clean-mode moderate –remove-invalid-genes -a core”) and, using mafft in Panaroo, an alignment suitable for phylogenetic analysis. We picked two of the most common antimicrobial resistance genes in our material (*lnuA* and *blaZ*) and created phylogenetic trees of these genes to illustrate the phylogenetic relationship of the resistance genes across isolates and species. For the phylogenetic trees of antimicrobial resistance genes, we also used mafft in Panaroo for gene alignment. All phylogenetic trees were then created using IQtree 2.0.3 with core gene alignment (and options “–safe -T AUTO -B 1000 -alrt 1000 -m MFP”). The graphic representation of the phylogenetic trees was done with iTOL (Letunic and Bork, 2019).

### Identification of Antimicrobial Resistance Genes

Identification of antimicrobial resistance genes was performed with ResFinder 4.1 (Center for Genomic epidemiology, Technical University of Denmark) (Zankari et al., 2017; Bortolaia et al., 2020). Prokka 1.131.3 was used for detection of the multidrug efflux pump gene *norA*.

### Identification of Virulence Genes

Identification of virulence genes was done by using VirulenceFinder 2.0 (Center for Genomic Epidemiology, Technical University of Denmark) (Joensen et al., 2014) and a tblastn 2.10.1 search against a published dataset (Naushad et al., 2019). The dataset by Naushad et al. (2019) was used to complement the VirulenceFinder program which, unexpectedly, returned very few results from the NAS species. The tblastn search of the proteins in the Naushad et al. (2019) dataset was set upwith a minimum high-scoring segment pair (HPS) coverage >90, a minimum e-value of 10^–5^. Ha scores were computed for all matches as described by Naushad et al. (2019). We kept only the hits with the highest Ha score and the highest percentage identity. Two proteins sequences were not identified in the database by Naushad et al. (2019): the phenol soluble modulin mec (PSMmec) and toxic shock syndrome toxin (tsst). For the PSMmec we used the record with GenBank accession number AIU84051.1, while the record for tsst from the database by Naushad et al. (2019) (accession number YP_415862) had been replaced by accession number WP_001035596.1, 100% identical to the previous record over its full length. Both records were found searching the NCBI (National Center for Biotechnology Information) website (^1^ accessed January 2021).

## Results

### Staphylococcal Isolates

**FIGURE 2:**
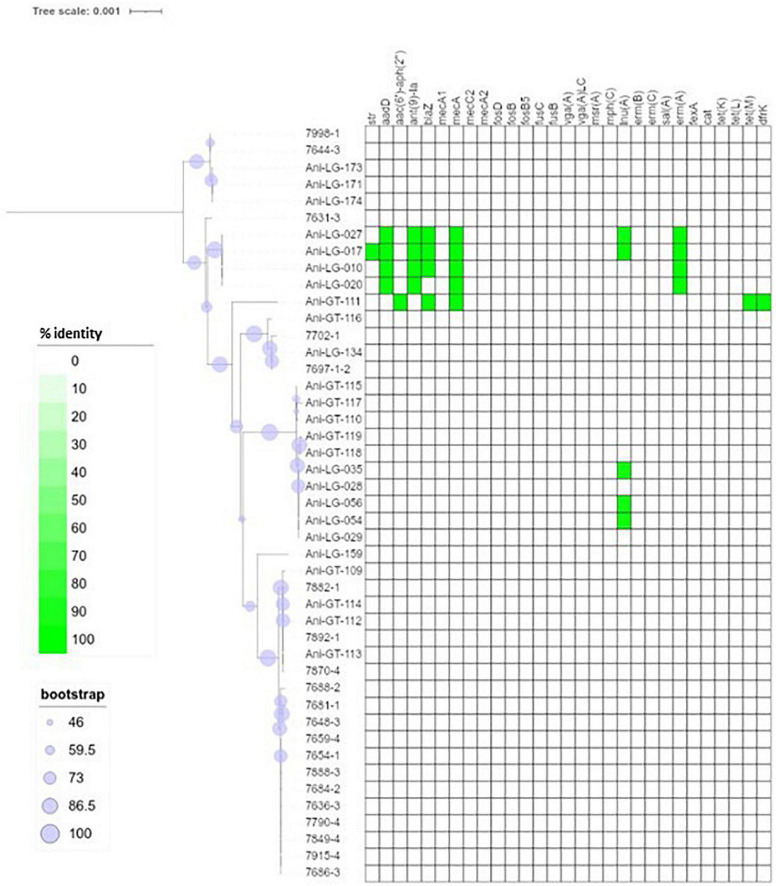
Phylogeny and antimicrobial resistance genes of *Staphylococcus aureus*. Presence of antimicrobial resistance gene is indicated with green square with percent identity indicated by color shades according to the scale.

Of the 95 isolates selected for WGS, results from 45 *S. aureus* and 48 NAS were included for further analysis. One *S. arlettae* and one *S. epidermidis* had to be rejected due to poor quality of the samples. The NAS species were divided into five different clades according to the phylogenetic analysis published by Naushad et al. (2016). NAS isolates were distributed in species and clades as follows; clade A was represented by *S. sciuri* (*n* = 5) and *S. vitulinus* (*n* = 1). Clade B was represented by *S. chromogenes* (*n* = 6) and *S. hyicus* (*n* = 1). Clade C consisted of *S. simulans* (*n* = 2). Clade D was represented by *S. devriesei* (*n* = 2), *S. epidermidis* (*n* = 7), *S. haemolyticus* (*n* = 9), *S. hominis* (*n* = 4) and *S. warneri* (*n* = 2). Clade E was represented by *S. arlettae* (*n* = 1), *S. auricularis* (*n* = 1), *S. cohnii* (*n* = 2), *S. equorum* (*n* = 2), *S. saprophyticus* (*n* = 1), and *S. xylosus* (*n* = 2). Species and clade distribution are also presented in Tables 1–7. Figure 1 shows the phylogeny of the NAS isolates, confirming that they do separate according to the different clades. Figure 2 shows the phylogeny of the *S. aureus* isolates.

**TABLE 1:** Distribution of virulence genes involved in adherence in all species.

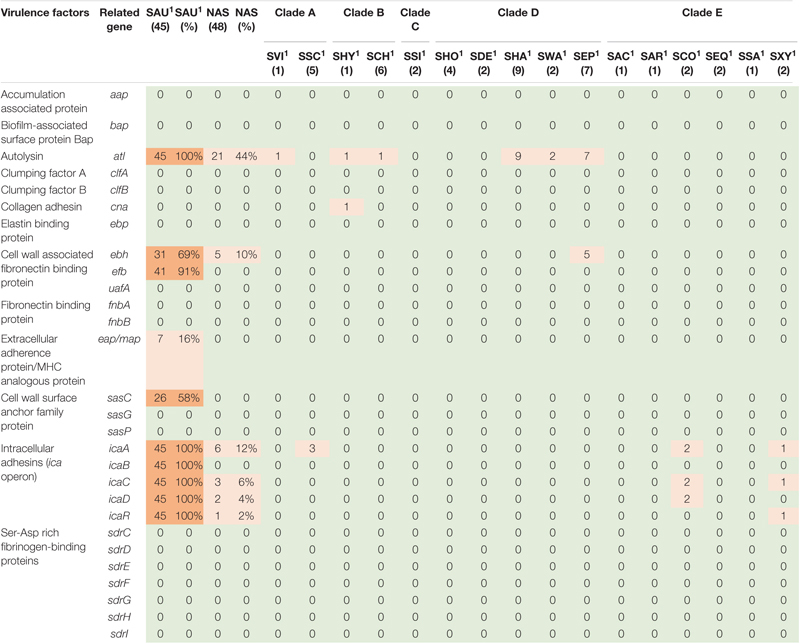

*NAS are shown according to clade. Green color indicates no virulence genes, light orange indicates the presence of virulence genes. For columns with S. aureus and total NAS dark orange indicates virulence gene present in over 50% of isolates.*

*^1^Species abbreviations: SAU, S. aureus; SVI, S. vitulinus; SSC, S. sciuri; SHY, S. hyicus; SCH, S. chromogenes; SSI, S. simulans; SHO, S. hominis; SDE, S. devriesei; SHA, S. haemolyticus; SWA, S. warneri; SEP, S. epidermidis; SAC, S. auricularis; SAR, S. arlettae; SCO, S. cohnii; SEQ, S. equorum; SSA, S. saprophyticus; SXY, S. xylosus.*

**TABLE 2:** Distribution of exoenzyme virulence genes in all species.

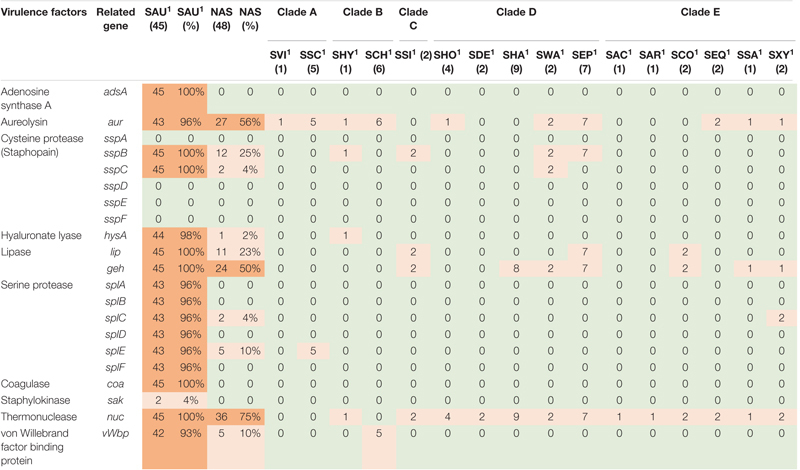

*NAS are shown according to clade. Green color indicates no virulence genes, light orange indicates the presence of virulence genes. For columns with S. aureus and total NAS dark orange indicates virulence gene present in over 50% of isolates.*

*^1^Species abbreviations: SAU, S. aureus; SVI, S. vitulinus; SSC, S. sciuri; SHY, S. hyicus; SCH, S. chromogenes; SSI, S. simulans; SHO, S. hominis; SDE, S. devriesei; SHA, S. haemolyticus; SWA, S. warneri; SEP, S. epidermidis; SAC, S. auricularis; SAR, S. arlettae; SCO, S. cohnii; SEQ, S. equorum; SSA, S. saprophyticus; SXY, S. xylosus.*

**TABLE 3:** Distribution of virulence genes involved in host immune evasion in all species.

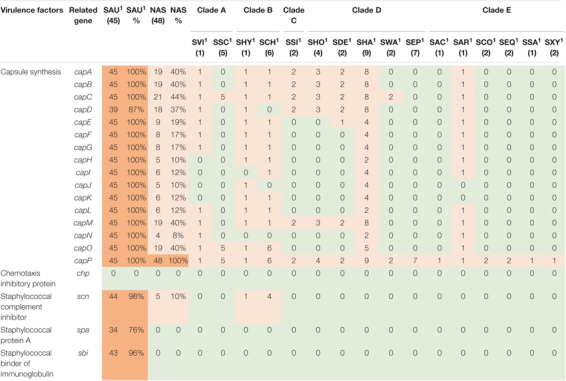

*NAS are shown according to clade. Green color indicates no virulence genes, light orange indicates the presence of virulence genes. For columns with S. aureus and total NAS dark orange indicates virulence gene present in over 50% of isolates.*

*^1^Species abbreviations: SAU, S. aureus; SVI, S. vitulinus; SSC, S. sciuri; SHY, S. hyicus; SCH, S. chromogenes; SSI, S. simulans; SHO, S. hominis; SDE, S. devriesei; SHA, S. haemolyticus; SWA, S. warneri; SEP, S. epidermidis; SAC, S. auricularis; SAR, S. arlettae; SCO, S. cohnii; SEQ, S. equorum; SSA, S. saprophyticus; SXY, S. xylosus.*

**TABLE 4:** Distribution of virulence genes involved in iron uptake and metabolism in all species.

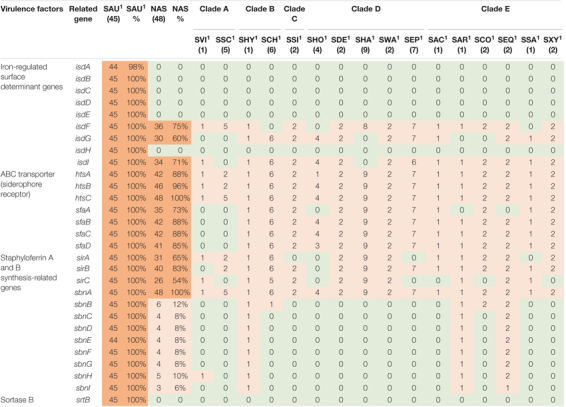

*NAS are shown according to clade. Green color indicates no virulence genes, light orange indicates the presence of virulence genes. For columns with S. aureus and total NAS dark orange indicates virulence gene present in over 50% of isolates.*

*^1^Species abbreviations: SAU, S. aureus; SVI, S. vitulinus; SSC, S. sciuri; SHY, S. hyicus; SCH, S. chromogenes; SSI, S. simulans; SHO, S. hominis; SDE, S. devriesei; SHA, S. haemolyticus; SWA, S. warneri; SEP, S. epidermidis; SAC, S. auricularis; SAR, S. arlettae; SCO, S. cohnii; SEQ, S. equorum; SSA, S. saprophyticus; SXY, S. xylosus.*

**TABLE 5:** Distribution of toxin, type IIV secretion and phenol-soluble modulin genes in all species.

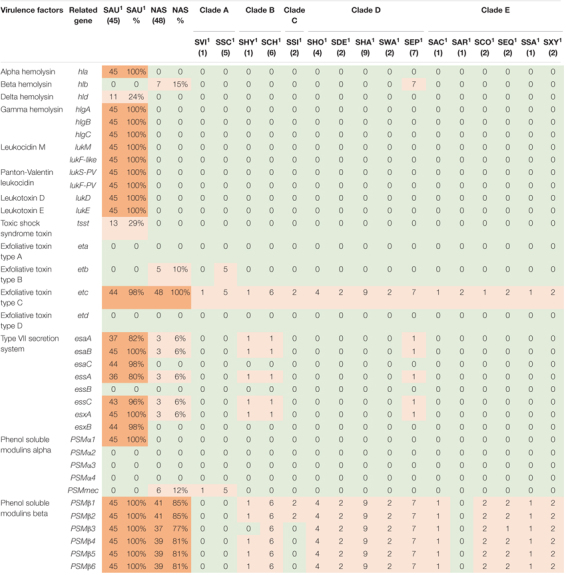

*NAS are shown according to clade. Green color indicates no virulence genes, light orange indicates the presence of virulence genes. For columns with S. aureus and total NAS dark orange indicates virulence gene present in over 50% of isolates.*

*^1^Species abbreviations: SAU, S. aureus; SVI, S. vitulinus; SSC, S. sciuri; SHY, S. hyicus; SCH, S. chromogenes; SSI, S. simulans; SHO, S. hominis; SDE, S. devriesei; SHA, S. haemolyticus; SWA, S. warneri; SEP, S. epidermidis; SAC, S. auricularis; SAR, S. arlettae; SCO, S. cohnii; SEQ, S. equorum; SSA, S. saprophyticus; SXY, S. xylosus.*

**TABLE 6:** Distribution of exotoxin genes in all species.

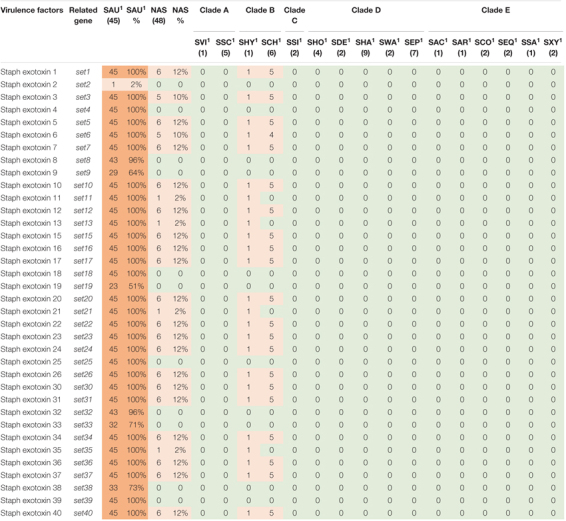

*NAS are shown according to clade. Green color indicates no virulence genes, light orange indicates the presence of virulence genes. For columns with S. aureus and total NAS dark orange indicates virulence gene present in over 50% of isolates.*

*^1^Species abbreviations: SAU, S. aureus; SVI, S. vitulinus; SSC, S. sciuri; SHY, S. hyicus; SCH, S. chromogenes; SSI, S. simulans; SHO, S. hominis; SDE, S. devriesei; SHA, S. haemolyticus; SWA, S. warneri; SEP, S. epidermidis; SAC, S. auricularis; SAR, S. arlettae; SCO, S. cohnii; SEQ, S. equorum; SSA, S. saprophyticus; SXY, S. xylosus.*

**TABLE 7:** Distribution of enterotoxin genes in all species.

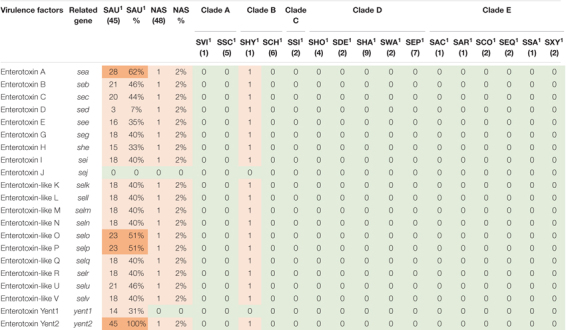

*NAS are shown according to clade. Green color indicates no virulence genes, light orange indicates the presence of virulence genes. For columns with S. aureus and total NAS dark orange indicates virulence gene present in over 50% of isolates.*

*^1^Species abbreviations: SAU, S. aureus; SVI, S. vitulinus; SSC, S. sciuri; SHY, S. hyicus; SCH, S. chromogenes; SSI, S. simulans; SHO, S. hominis; SDE, S. devriesei; SHA, S. haemolyticus; SWA, S. warneri; SEP, S. epidermidis; SAC, S. auricularis; SAR, S. arlettae; SCO, S. cohnii; SEQ, S. equorum; SSA, S. saprophyticus; SXY, S. xylosus.*

### Antimicrobial Resistance Genes

Figure 1 shows the distribution of antimicrobial resistance genes in all NAS isolates, in which the NAS are shown according to their clade. Figure 2 shows the distribution of antimicrobial resistance genes in all *S. aureus* isolates.

**FIGURE 3:**
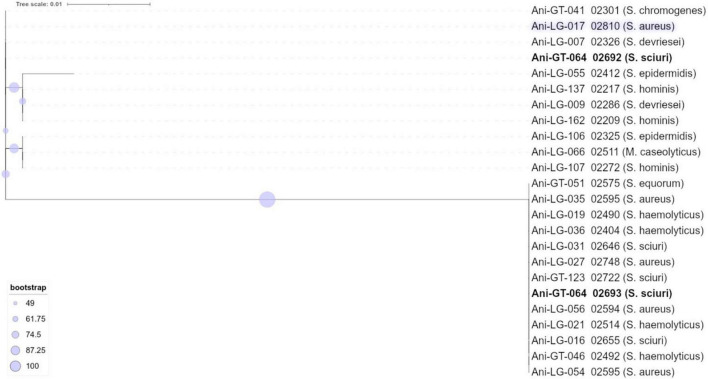
Phylogeny of lincosamide resistance gene *lnuA* found in staphylococci.

Lincosamide resistance gene *lnuA* was present in 17 isolates from seven different NAS species. Most NAS isolates originated from clade D (*n* = 11). The gene was also present in five *S. aureus* isolates. Figure 3 shows the phylogeny of the *lnuA* genes, demonstrating that several species carried phylogenetically similar *lnuA* genes. Macrolide resistance genes were found in 17 different isolates, with some isolates (Ani-GT-049, Ani-LG-025, Ani-LG-026 and Ani-LG-125) containing multiple macrolide resistance genes. The *erm* genes were present in four *S. aureus* isolates and eight NAS isolates, of which five were of clade D. The four *S. aureus* isolates contained the *ermA* gene, while the *ermB* and *ermC* genes were detected uniquely in the NAS species. The *mphC* gene was present only in NAS species, most originating from clade E (*n* = 4). The *msrA* gene was present in species from clade D and E.

**FIGURE 4:**
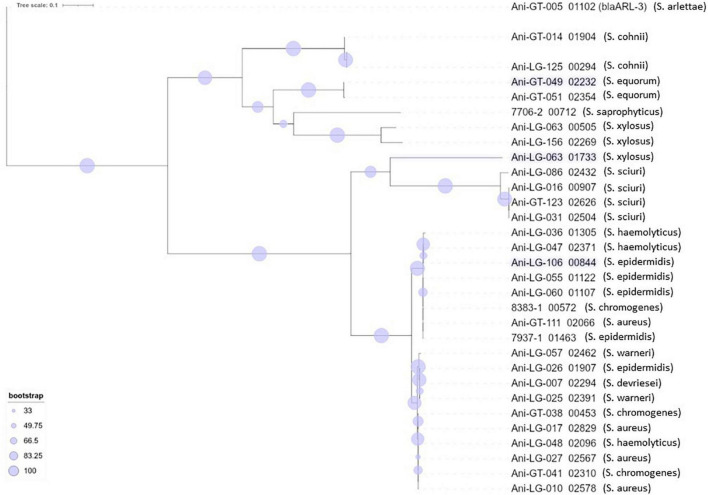
Phylogeny of penicillin resistance gene *blaZ* found in staphylococci. The novel penicillin resistance gene *bla*_ARL_ found in *Staphylococcus arlettae* (Andreis et al., 2017) is included for phylogenetic comparison.

The penicillin resistance gene *blaZ* was found in 19 isolates, both *S. aureus* and NAS, with most NAS isolates originating from clade D (*n* = 11). Figure 4 shows the phylogeny of the *blaZ* genes. One *S. xylosus* isolate contained up to two phylogenetically distinct *blaZ* genes. The betalactam resistance gene *mecA* was present in seven isolates, *S. aureus* (*n* = 5), and *S. epidermidis* (*n* = 1), and *S. haemolyticus* (*n* = 1) from clade D. Of these, four *S. aureus* and one *S. haemolyticus* also carried *blaZ*. All other resistance genes detected in *S. aureus* were detected in the *mecA* positive isolates, except for three non-*mecA S. aureus* isolates containing only the *lnuA* gene. Other variants of the *mec* genes were also detected in some isolates. All *S. sciuri* isolates (*n* = 5) carried the *mecA1* gene, the only *S. vitulinus* carried the *mecA2* and one of the two *S. xylosus* carried *mecC2*.

Of the tetracycline resistance genes detectable by ResFinder, the *tetK* gene was present in 11 NAS isolates from clade A, D and E, and the *tetL* gene was present in three NAS species from clade B and D. The *tetM* gene was present in one *S. aureus* isolate only.

The aminoglycoside resistance gene *aadD* was identified in four *S. aureus* isolates and four NAS isolates consisting of the following species: *S. chromogenes*, *S. haemolyticus*, *S. sciuri*, and *S. warneri*. One *S. aureus* isolate carried the aminoglycoside resistance gene *aac-aph*. The aminoglycoside resistance gene *str* was detected in nine NAS isolates from clade A, D, and E.

Overall, in NAS, many antimicrobial resistance genes were found in isolates from clade D and most isolates in this clade carried one to three resistance genes. In one *S. chromogenes* isolate (clade B), seven resistance genes were detected, including *lnuA, blaZ, ermB, aadD, tetL, dfrK*, and *fexA*. In addition, one *S. equorum*, one *S. cohnii* (both clade E), and one *S. warneri* (clade D) carried five resistance genes, with aminoglycoside, macrolide and tetracycline resistance genes detected in all three isolates. No antimicrobial resistance genes were identified in only nine of the 48 NAS isolates, which originated from clade B, C, D, and E. On the other hand, no antimicrobial resistance genes were detected in the majority of the *S. aureus* isolates (37/45). Five *S. aureus* isolates carried three to six resistance genes, the *mecA* gene was detected in all five.

Using Prokka, the major facilitator superfamily multidrug efflux transporter gene *norA* was detected in all isolates, both *S. aureus* and NAS.

### Virulence Genes Detected by VirulenceFinder

**FIGURE 5:**
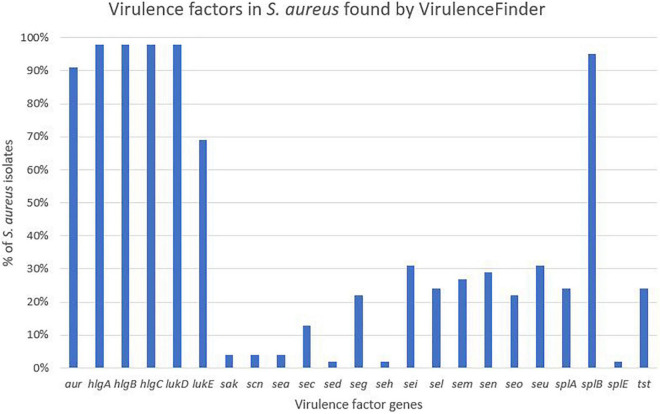
Distribution of virulence genes in *Staphylococcus aureus* found by VirulenceFinder.

Virulence genes detected by VirulenceFinder (Center for Genomic Epidemiology, Technical University of Denmark) in the *S. aureus* isolates are shown in Figure 5. The *hlgA*, *hlgB*, and *hlgC* genes encoding the gamma hemolysins A, B, and C, and the *lukD* gene coding for a leukotoxin, were the most commonly detected virulence genes in *S. aureus* and detected in 44/45 *S. aureus* isolates. The *splB* gene, encoding a serine protease, and *aur*, encoding aureolysin, were detected in 43 and 41 *S. aureus* isolates, respectively. The least frequent virulence genes identified by VirulenceFinder were *sak*, *scn*, and *sea*, encoding staphylokinase, staphylococcal complement inhibitor and enterotoxin A, respectively, found in two *S. aureus* isolates only (Ani-LG-017 and Ani-LG-027), and the *sed* and *seh* genes coding for enterotoxin D and H, were detected in one *S. aureus* isolate each.

Except for the detection of the *ACME* gene in four *S. epidermidis* isolates, the VirulenceFinder did not detect any other virulence genes in the NAS isolates.

### Putative Virulence Factors Based on Dataset by Naushad et al., 2019

#### Virulence Factors Involved in Adherence

Based on the dataset by Naushad et al. (2019), 28 virulence-related genes involved in adherence were screened for. The genes are listed, and results are summarized in Table 1. The *atl* gene was present in 21 NAS isolates and in six of the 16 NAS species from clade A, B, and D, as well as in all *S. aureus* isolates. The *icaA* of the *ica* operon was present in six NAS isolates from clade A and E. The *icaC, icaD*, and *icaR* were detected in three, two and one NAS isolates respectively, from clade E. Regarding the *S. aureus* isolates, the *ica* operon was present in all isolates. Many of the adherence associated genes were not detected in either NAS isolates nor *S. aureus* isolates, including *aap, bap, clfA* and *clfB, ebp, uafA*, *fnbA* and *fnbB, sasG* and *sasp* and the Ser-Asp rich fibrinogen-binding proteins.

#### Exoenzymes

Twenty-one different exoenzyme genes were searched. The genes are listed, and results are summarized in Table 2. The *nuc* gene was present in 36 out of 48 NAS isolates in species from clade B, C, D, and E. Only one *S. hyicus* isolate was positive in clade B, while in clade C, D, and E all isolates were positive. The second most frequent exoenzyme genes in NAS were *aur* and *geh*, detected in 27 and 24 isolates, respectively. Among the serine proteases, *splC* and *splE* were detected in *S. xylosus* and *S. chromogenes*, respectively and *vWpb* was found in *S. chromogenes*. Many of the exoenzymes were not detected in NAS species. For *S. aureus*, 93% of all *S. aureus* isolates contained at least 16 exoenzyme genes.

#### Virulence Factors Involved in Host Immune Evasion

Twenty virulence genes associated with host immune evasion were searched. The genes are listed, and results are summarized in Table 3. The *chp*, *spa* and *sbi* genes were not detected in any of the NAS species. The *scn* gene was only identified in species from clade B. The *cap* genes were the most frequently detected host immune evasion genes. Based on Naushad et al. (2019) *cap* genes were considered present if either the *cap5* or *cap8* isoforms were detected. The *capP* was detected in all NAS isolates and five NAS species contained only this *cap* gene. Two *S. haemolyticus* isolates contained all 16 *cap* genes and the single *S. hyicus* and *S. arlettae* contained 14 *cap* genes. All *cap* genes except *capD* were present in all *S. aureus* isolates. The *scn*, *spa* and *sbi* genes were identified in 44, 34, and 43 *S. aureus* isolates, respectively. The *chp* gene was not detected in any of the *S. aureus* isolates.

#### Virulence Factors Involved in Iron Uptake and Metabolism

Twenty-nine iron uptake and metabolism genes were searched. The genes are listed, and results are summarized in Table 4. The ABC transporter genes and staphyloferrin A and B synthesis related genes were most frequently identified in NAS isolates, where *htsC* and *sbnA* were present in all NAS isolates. All genes associated with iron uptake and metabolism were detected in all *S. aureus* isolates.

#### Toxins, Type VII Secretion and Phenol-Soluble Modulin Genes

The genes in this category included 36 toxin genes from various categories. The genes are listed, and results are summarized in Table 5. The enterotoxins and staphylococcal exotoxins are described below and in Tables 6, 7. Most of the toxin genes in this category were not detected in the NAS species, except *etc* which was present in all NAS isolates, *hlb* present in all *S. epidermidis* isolates and *etb* present in all *S. sciuri* isolates. The type VII secretion genes *esaA*, *esaB*, *essA*, *essC*, and *esxA* were identified in three NAS isolates, namely one *S. chromogenes*, one *S. epidermidis* and the single *S. hyicus*. Among the PSMs, none of the PSMα were detected, while the PSMβ genes were present in all NAS species except the species in clade A where PSMmec was identified. As for the *S. aureus* isolates, all isolates contained *hla*, *hlgA, hlgB, hlgC*, and all leukocidin and leukotoxin genes. The *etc* gene was identified in 44 isolates and the *tsst* and *hld* genes were identified in 13 and 11 *S. aureus* isolates, respectively, while none of the *S. aureus* isolates contained *hlb*, *eta, etb* or *etd*. Most of the type VII secretion system genes were frequently detected in *S. aureus* isolates, except *essB* that was not present in any of the isolates. Of the PSMs, *PSM*α*1*, and *PSM*β*1* to *PSM*β*6* were identified in all *S. aureus* isolates, the remaining PSMs were not identified.

Thirty-six staphylococcal exotoxins (SETs) and 21 enterotoxins were searched. Except for the species in clade B, *set* genes were not detected in any NAS species. The enterotoxins were only identified in the single *S. hyicus* of clade B, this isolate contained all enterotoxin genes except *sej* and *yent1*. In the *S. aureus* isolates most *set* genes were present in all isolates. *set9, set19, set 32, set33*, and *set38* were frequently detected, while *set2* was only present in one *S. aureus* isolate. The *yent2* gene was detected in all *S. aureus* isolates. The second most frequently identified enterotoxin genes were *sea*, *selo*, and *selp*, present in 28, 23, and 23 *S. aureus* isolates. The *sej* gene was not detected in any *S. aureus* isolates.

#### Virulence Potential

Virulence potential was defined as the total number of virulence genes in an isolate, where all genes were equally weighted (Naushad et al., 2019). *Staphylococcus aureus* had the highest virulence potential and carried on average 140 virulence genes. The NAS isolates, disregarding species, carried on average 28 virulence genes. The highest virulence potential in NAS was detected in *S. chromogenes* and *S. hyicus* (both clade B). *Staphylococcus chromogenes* isolates contained on average 44 virulence genes, and, except the single *S. hyicus* isolate, was the only NAS species in which staphylococcal exotoxins were detected. The single *S. hyicus* isolate contained 98 virulence genes, carrying both exotoxins and enterotoxins. The lowest virulence potential was found in *S. sciuri* and *S. hominis* with, on average, 13 and 21 virulence genes in total, respectively.

## Discussion

This study of virulence and antimicrobial resistance genes in 93 whole-genome sequenced NAS and *S. aureus* isolates from bovine milk samples of European origin adds new data to the current sparse information of the genetic basis for both antimicrobial resistance and virulence factors in bovine staphylococci. WGS confirmed the species distribution previously determined with Maldi Tof-MS of the isolate collection of 45 *S. aureus* and 48 NAS isolates of different species (Fergestad et al., 2021). In total, we determined the presence of 191 staphylococcal virulence genes and 25 antimicrobial resistance genes. When discussing our results, it must be taken into account that the whole-genome sequenced isolates are not a random assemblage of bovine staphylococci, but they are selected from a previous study according to the described criteria. Nevertheless, one main finding is that our description of the virulence gene contents of the 48 NAS isolates coincides to a large extent with the findings of the so far most comprehensive study of virulence genes in a collection of Canadian bovine NAS based on WGS data.

The emergence and spread of antimicrobial resistance genes are of great concern to society, including animal food production and the dairy industry. The antimicrobial resistance genes that are detected by ResFinder 4.1 were frequently observed in our collection of isolates representing 16 NAS species, coinciding with other reports of occurrence of such genes distributed among several NAS species (Nobrega et al., 2018). This finding of an array of resistance genes in a diversity of NAS species is supporting the hypothesis that these bacteria can act as a potential reservoir for resistance properties (Otto, 2013; Becker et al., 2014).

We found several isolates from different staphylococcal species harboring phylogenetically similar *lnuA* genes. This gene, encoding a lincosamide nucleotidyltransferase that confers resistance to lincosamides, has previously been found in both *S. aureus* and NAS of bovine origin (Lüthje and Schwarz, 2006; Li et al., 2015). The gene is often found on plasmids, which could promote horizontal transfer of the gene (Lüthje et al., 2007). Studies have shown that the nucleotide sequence of *lnuA* is more conserved than the surrounding plasmid sequences and the conserved gene has been found in several different plasmid backbones, suggesting that the gene is also exchanged via interplasmid recombinational events (Lüthje et al., 2007; Wassenaar et al., 2016). This may contribute to explaining our finding of phylogenetically similar *lnuA* genes in several different staphylococcal species.

Resistance to betalactam antimicrobials is commonly reported in staphylococci. The *blaZ* gene encodes a penicillinase (or betalactamase) conferring penicillin resistance by hydrolyzing the betalactam ring and inactivating the drug (Zhang et al., 2001). The gene is usually either plasmid- or chromosomally encoded (Olsen et al., 2006). Penicillin resistance is prevalent in *S. aureus* of both human and bovine origin (Olsen et al., 2006) and betalactamase production is the most prevalent mechanism of betalactam resistance in NAS (Nobrega et al., 2018). In consistence with these observations, we found several isolates of different staphylococcal species carrying the *blaZ* gene. The phylogenetic tree of the gene also showed several phylogenetically different sequences. A high number of different *blaZ* sequence types has previously been shown in staphylococci of bovine origin, as well as a very low similarity between plasmid- and chromosomally encoded *blaZ* genes which, in a study by Olsen et al. (2006), separate into two phylogenic clusters, leading to the conclusion that exchange of *blaZ* between plasmid and chromosome and between strains are rare events (Olsen et al., 2006). Our *blaZ* phylogenetic tree (Figure 4) also display two separate clusters. Interestingly, we observed one *S. xylosus* isolate with two distinct *blaZ* genes, one from each branch/cluster.

We identified five *mecA* positive *S. aureus* isolates from Belgium, which were selected to be included. Four isolates were from the same herd and epidemiologically related. The *mecA-*positive *S. aureus* isolates differed from the rest of the *S. aureus* isolates, with a larger content of antimicrobial resistance genes, but due to their epidemiological relation this result must be interpreted with caution. A higher frequency of antimicrobial resistance in methicillin-resistant *S. aureus* (MRSA) compared to methicillin-susceptible *S. aureus* (MSSA) has, however, been shown in human isolates (Thompson and Brown, 2014). Two NAS isolates also carried the *mecA* gene, one being *S. epidermidis* and one *S. haemolyticus*. *Staphylococcus epidermidis* and *S. haemolyticus* have previously been shown to have a higher prevalence of *mecA* compared to other staphylococcal species (McManus et al., 2015; Nobrega et al., 2018). In addition, all *S. sciuri* harbored the *mecA1* gene, while the *mecA2* and *mecC2* were found in one *S. vitulinus* and one *S. xylosus*, respectively. There is support for a theory suggesting that *mecA* evolved from native *mec* genes in species of the *S. sciuri* group (Couto et al., 1996; Zhou et al., 2008; Antignac and Tomasz, 2009). The *mecA1* gene, thought to be ubiquitous in *S. sciuri*, is believed to be the most ancestral form of *mecA* and shares 85% nucleotide identity with *S. aureus mecA*, while the *mecA2* of *S. vitulinus* is an intermediate form with 94% homology (Miragaia, 2018). However, neither *mecA1* nor *mecA2* generally confers methicillin resistance (Couto et al., 1996; Wu et al., 1996; Miragaia, 2018). The *mecC2* gene, a *mecC* allotype with 92.9% identity with the *S. aureus mecC*, has previously been described in *S. saprophyticus* (Małyszko et al., 2014).

Several staphylococcal species in our study also harbored the macrolide resistance genes *ermA-C*. In the *S. aureus* isolates only *ermA* was found, while *ermC* and to some extent *ermB* were found in the NAS species. The *ermA*-positive *S. aureus* isolates were also *mecA* positive, labeling them as MRSA. This concurs with the results of a previous study that found *ermA* to be more prevalent than *ermB* and *ermC* in MRSA isolates and the *ermC* to be more prevalent than *ermA* and *ermB* in NAS (Lina et al., 1999).

Several studies have supported the role of drug efflux in the development of antimicrobial resistance in *S. aureus* (DeMarco et al., 2007; Kwak et al., 2013; Santos Costa et al., 2015). Especially multidrug efflux pumps are of interest, being able to remove several chemically different substances and often linked to multidrug resistant phenotypes (Piddock, 2006; Poole, 2007). The major facilitator superfamily multidrug efflux transporter norA is the best studied efflux system in *S. aureus* and is associated with resistance to fluoroquinolones and several antiseptics and disinfectants (Costa et al., 2018). The gene is believed to be a part of the core genome of *S. aureus* (Costa et al., 2018), while Nobrega et al. (2018) reported the *norA* gene in 91% of NAS isolates. Consequently, our finding of the *norA* gene in all isolates, both in *S. aureus* and NAS, is coherent with these previous data.

Many antimicrobial resistance genes were detected in NAS isolates from clade D. Multidrug resistant *S. epidermidis* and *S. haemolyticus*, both from clade D, have previously been isolated from both humans and animals (Anthonisen et al., 2002; Taponen et al., 2016; Lee et al., 2018; Nobrega et al., 2018; Fergestad et al., 2021). However, both the preselection of isolates for whole-genome sequencing and the fact that clade D was the clade with the highest number of isolates, might be skewing the observed distribution of resistance genes in our material.

Regarding virulence there are many factors involved in colonization, infection, and bacterial survival. Adhesion is one of the first steps leading to colonization and infection and the process is also needed for biofilm formation. We analyzed the genome data for the presence of 28 adherence and biofilm associated virulence factor genes. The *atl* gene, most frequently observed in NAS, is involved in biofilm formation through initiating adherence, followed by production of polysaccharide intracellular adhesins encoded by the *ica*-operon, forming a polysaccharide-based biofilm (Naushad et al., 2019). Deviating results about the distribution of *ica* genes in bovine NAS have been reported (Piessens et al., 2012; Tremblay et al., 2013), and a study concerning the *icaA* genes of nine food-related NAS species showed considerable sequence diversity between strains of the same species (Møretrø et al., 2003). Diverging sequences could explain differences in the detection of *ica* genes. Our observation of the frequent detection of *icaA* followed by *icaC* and *icaD* resembles the findings of Naushad et al. (2019) who found *icaC*, followed by *icaA* and *icaD* to be most frequent. The whole *ica* operon were detected in all *S. aureus* isolates in this study, similar to findings from other studies (Melchior et al., 2009, 2011). In human staphylococcal strains, biofilms associated with the *ica*-operon are often related to infections in foreign devices, leading to the hypothesis that the *ica* genes in bovine isolates could play a role outside the udder, by promoting adhesion to abiotic surfaces, such as milking equipment (Melchior et al., 2011).

The production of exoenzymes further facilitates colonization and infection. Following adhesion, exoenzymes contributes to disable the host immune system, damage tissue and acquire nutrients (Tam and Torres, 2019). The thermonuclease gene *nuc* was the most frequently observed exoenzyme gene in NAS species in our study, consistent with recent studies (Åvall-Jääskeläinen et al., 2018; Naushad et al., 2019). The *aur* gene and both lipase gene *geh* and *lip* were frequently observed in the NAS group, however, the lipase genes only in clades C, D and E. These results also concur with the findings by Naushad et al. (2019), who found these genes to be frequently distributed in clades B to E. In addition, similar to Naushad et al. (2019), we detected *vWbp* in *S. chromogenes*, which could explain the variable coagulase test results for this species (Dos Santos et al., 2016). A large proportion of the *S. aureus* isolates in this study contained most of the sought exoenzymes. It is well known that *S. aureus* can produce a vast variety of exoenzymes, degrading host and bacterial molecules to escape the host immune system and gain nutrients, contributing to the success of the pathogen (Tam and Torres, 2019).

Staphylococci, especially *S. aureus*, have several host immune evasion virulence factors, such as genes allowing production of capsular polysaccharides, enabling bacterial survival and dissemination by hindering phagocytosis and increasing virulence (Kuipers et al., 2016). The cap5A-P and cap8A-P are prevalent in *S. aureus* of bovine origin (Salimena et al., 2016). In our study, *capP* was present in all NAS isolates, while *capA-D*, *capM* and *capO* were present in almost half of the NAS isolates. This deviates some from the findings of Naushad et al. (2019), who found *capM* to be most frequent and *capA-L* in low frequencies. Except *capD*, all *cap* genes were present in all *S. aureus* isolates, however, *capD* was also present in most *S. aureus* isolates.

In addition to the capsular genes, staphylococci can produce other important immune evasion virulence factors, such as *chp*, *scn*, *spa* and *sbi*. The chemotaxis inhibitory protein (encoded by *chp*) and staphylococcal complement inhibitor (encoded by *scn*) are mostly believed to be found in staphylococci from human sources (Verkaik et al., 2011). Consistent with this finding, we did not detect the *chp* gene in any isolate. However, in accordance with the study by Naushad et al. (2019), we identified *scn* in species of clade B. The *scn* gene was also detected in all except one of the *S. aureus* isolates.

Iron is an essential micronutrient involved in several metabolic processes, vital for bacterial survival and growth (Sheldon and Heinrichs, 2015). During infection, the host withdraws free iron from body fluids to suppress pathogens (Haley and Skaar, 2012; Sheldon and Heinrichs, 2015). Mechanisms to acquire iron in a situation where the supply is scarce are well studied in *S. aureus*, who can take up iron directly from molecules using *isd* genes and produce siderophores along with surface transporters (Sheldon and Heinrichs, 2015). We did indeed detect all iron uptake and metabolism genes, except *isdA*, in all *S. aureus* isolates. In the NAS species, however, ABC transporter and staphyloferrin A genes were more frequently detected, compared to *isd* genes and staphyloferrin B genes. This is in accordance with the study by Naushad et al. (2019), and supports their hypothesis that staphyloferrin A production is the principal mechanism for iron acquisition in NAS.

The production of toxins is another important determinant of virulence in staphylococci, especially in *S. aureus*. These toxins, such as cytotoxins (hemolysins, leukotoxins, and leukocidins) and superantigens [enterotoxins, exfoliative toxins and toxic shock syndrome toxins (TSST)], promotes inflammation and leukocyte cell death (Tam and Torres, 2019). Of the cytotoxins, we detected *hlb*, encoding beta hemolysin, in all *S. epidermidis* isolates (clade D), similar to Naushad et al. (2019), who found *hlb* to be the most frequent hemolysin. Surprisingly, we did not detect *hlb* in any of the *S. aureus* isolates, whereas *hla* was present in all isolates. This contrasts the findings from a study on bovine and humans *S. aureus* isolates where *hlb* appeared more common in bovine *S. aureus* isolates, while *hla* was more prevalent in the human isolates (Aarestrup et al., 1999). Similar to the study by Naushad et al. (2019), we did not detect any leukocidin genes or leukotoxin genes in the NAS isolates. Åvall-Jääskeläinen et al. (2018) found *lukD* in one *S. simulans* isolate, however, none of the other leukocidin or leukotoxin genes were detected in NAS in their study. All the *S. aureus* isolates in our study contained all genes for leukocidins and leukotoxins, including *lukS-PV* and *lukF-PV*. This resembles findings from another study on bovine *S. aureus* isolates that found leukocidin and leukotoxin genes in most isolates. However, that study did not detect any isolates carrying *lukS-PV* (Yamada et al., 2005). The Panton Valentine Leukocidin (PVL) genes are believed to be restricted to human strains of *S. aureus* (Vrieling et al., 2016) and it was surprising to find these genes in all our bovine *S. aureus* isolates. It is possible that this result appeared due to the method of similarity search, as there is a possibility for detecting genes that are similar to the gene in question, although not being the same gene. This could explain our results, as many of the *lukS-PV* and *lukF-PV* genes in our study had the same percentage identity in several *S. aureus* isolates, possibly indicating that there are sequence similarities between the genome and the genes, although the genes in question are not actually present. However, this warrants further investigation. The PVL genes have been reported in a few NAS isolates of bovine origin in India (Mahato et al., 2017). Of the exfoliative toxin genes, we detected *etc* in all NAS isolates and *etb* in all *S. sciuri* isolates. This contrasts the findings of Naushad et al. (2019) who found *eta* in all isolates of three NAS species and *etb* in a few isolates of *S. agnetis* and Åvall-Jääskeläinen et al. (2018) who also found *etb* in *S. agnetis*. However, the latter did not test for *eta, etc*, and *etd*. We did not have any *S. agnetis* in our collection, and it is unknown whether there are geographical differences between the distribution of the different exfoliative toxin genes or if there are other factors affecting our results. Of the exfoliative toxin genes, only the *etc* gene was detected in *S. aureus* isolates. The lack of *eta* and *etb* has been shown previously by Haveri et al. (2007), however, these were the only two exfoliative toxin genes included in this study. The *eta* gene has been detected in a few *S. aureus* isolates from bovines (Hayakawa et al., 2001).

Phenol soluble modulins (PSM) are also involved in the killing of leukocytes and can act synergistically with leukocidins (Hongo et al., 2009), contributing to the leukotoxicity of *S. aureus*, as well as being involved in biofilm formation (Otto, 2014; Vrieling et al., 2016). The PSMs are considered major determinants of the virulence of *S. aureus* and α-type PSMs are thought to be more aggressive than β-type PSMs (Otto, 2014; Naushad et al., 2019). Being encoded on the core genome, the PSMs are present in virtually all staphylococci (Otto, 2014). We detected β-type PSMs genes, encoding the least aggressive PSMs, in most NAS species, but not in species of clade A and the *S. arlettae* isolate (clade E). This concurs with the results reported by Naushad et al. (2019). However, in the species of clade A (*S. sciuri* and *S. vitulinus*) we detected the *PSMmec* gene. This is the only exception to the core genome-encoded PSMs, as the *PSMmec* is often found on the SCC*mec* cassette carrying the *mecA* genes conferring methicillin resistance (Qin et al., 2016). The isolates in our study in which the *PSMmec* was found did indeed harbor *mec* genes, as the *S. sciuri* isolates harbored the *mecA1* and the *S. vitulinus* isolate harbored the *mecA2* gene. The *PSMmec* has been identified in *S. vitulinus* carrying SCC*mec* previously (Monecke et al., 2012). In *S. aureus PSM*α*1*, as well as *PSM*β*1-6* were detected in all isolates. The finding of α-type PSM in *S. aureus* and not in NAS can be due to the aggressive potential of *S. aureus*, as the α-type PSM are considered more aggressive and this type of PSMs is mostly associated with *S. aureus* (Wang et al., 2007). The fact that we only detected *PSM*α*1* and not the other *PSM*α can be due to limitations in the method used (similarity search) and the short sizes of *PSM*α, as this often does not give meaningful results (Cheung et al., 2014; Otto, 2014).

Superantigens are responsible for much of the toxicity in staphylococci. They are robust toxins, resilient to heat, proteolysis and desiccation (Spaulding et al., 2013; Tam and Torres, 2019). Toxic shock syndrome toxin gene (*tsst*) was not detected in the NAS isolates in our study. This concurs with several previous studies (Xu et al., 2015; Mello et al., 2016; Naushad et al., 2019). The *tsst* gene is mostly associated with *S. aureus* and has been detected in bovine *S. aureus* previously (Artursson et al., 2016; Vaughn et al., 2020). We only detected staphylococcal exotoxins in species of clade B (*S. hyicus* and *S. chromogenes*), however, several exotoxins were present in all *S. aureus* isolates. We only detected enterotoxin genes in one *S. hyicus* isolates (clade B). Naushad et al. (2019) also detected enterotoxin genes only in species from clade B. However, unlike Naushad et al. (2019) we did not identify any enterotoxin genes in *S. chromogenes*. We also detected several enterotoxin genes in *S. aureus* isolates. The *S. aureus* enterotoxins can cause acute and severe food poisoning, making it important to avoid contamination of enterotoxin-producing *S. aureus* isolates throughout the food production chain (Nia et al., 2016). Raw milk and cheese made of unpasteurized milk are well-known food sources of food poisoning caused by *S. aureus*.

Regarding the total virulence gene content, *S. aureus* stands out from NAS with, on average, five times as many virulence genes as NAS, highlighting the large virulence potential of *S. aureus* and the limited virulence potential of NAS. When looking at the total number of virulence genes in NAS, *S. chromogenes* had a higher virulence potential than most other species, mostly due to the presence of exotoxins. The single *S. hyicus* isolate stood apart with the highest virulence potential, due to host immune evasion, exotoxin and enterotoxin genes. This concurs with the findings of Naushad et al. (2019) who also found the highest virulence potential in species from clade B, including *S. chromogenes* and *S. hyicus*. Virulence is dependent on context and is often characterized by an intricate interplay between the microorganism and the host, making it difficult to predict virulence based on virulence gene content alone (Balloux et al., 2018). Nor is it a given that the virulence genes are expressed in the microorganism, even if they are present (Chaves-Moreno et al., 2016), which further complicates the matter. Considering the apparent complexity of virulence and the limited knowledge on the subject in bovine NAS, further studies on the association between virulence and clinical impact of these species is important. As several studies indicates that *S. chromogenes* could have a greater impact on udder health (Supré et al., 2011; Fry et al., 2014; Valckenier et al., 2019) and both this study and Naushad et al. (2019) found *S. chromogenes* among those with the highest virulence potential, further studies of the virulence and pathogenesis of this species should be emphasized (De Buck et al., 2021).

Regarding the *S. aureus* isolates, the results from VirulenceFinder supported the results obtained using the database by Naushad et al. (2019). However, some genes were not detected as frequently with the VirulenceFinder, possibly due to stricter thresholds for identity in VirulenceFinder. In addition, the VirulenceFinder returned less virulence genes compared to the database from Naushad et al. (2019). This could be because VirulenceFinder contains less virulence genes compared to the database by Naushad et al. (2019), as the database was expanded by doing blast similarity search to identify genes. Although the similarity search method does identify true genes, there is a possibility for misinterpretations due to detection of similar, but not identical genes.

In conclusion, our data support the opinion that there are more antimicrobial resistance genes in NAS compared to *S. aureus*. Regarding virulence, our *S. aureus* isolates had a higher virulence potential compared to NAS, but there are also differences in virulence gene content within the NAS group, supporting the view that NAS should not be considered as one uniform group of bacteria.

## Data Availability Statement

The datasets presented in this study can be found in online repositories. The names of the repository/repositories and accession number(s) can be found below: https://www.ncbi.nlm.nih.gov/, PRJNA609060.

## Author Contributions

FT was in charge of the methodology, performed the methods used, and wrote sections of the manuscript. MF organized the databases, analyzed the results, and wrote the first draft of the manuscript. SD, AD, and YW contributed to the analysis of the results. All authors contributed to the conception and design of the study, the manuscript revision, and read and approved the submitted version.

## Conflict of Interest

The authors declare that the research was conducted in the absence of any commercial or financial relationships that could be construed as a potential conflict of interest.

## Publisher’s Note

All claims expressed in this article are solely those of the authors and do not necessarily represent those of their affiliated organizations, or those of the publisher, the editors and the reviewers. Any product that may be evaluated in this article, or claim that may be made by its manufacturer, is not guaranteed or endorsed by the publisher.

## Footnotes

1
https://www.ncbi.nlm.nih.gov/
