# Supplementary material for: Whole Genome Sequencing of Staphylococci Isolated From Bovine Milk Samples
Source: Front Microbiol. 2021 Dec 20;12:715851. doi: 10.3389/fmicb.2021.715851 (PMC8721127; doi:10.3389/fmicb.2021.715851)
Supplement: Supplementary file 1 [file Table_1.DOCX]

Supplementary table 1. Distribution of staphylococcal isolates based on species, antimicrobial resistance and country of origin.

| Species | Total | Ampicillin^1^ | Amoxi/  clav^1^ | Ciprofloxacin^1^ | Clindamycin^1^ | Erytromycin^1^ | Gentamicin^1^ | Linezolid^1^ | Penicillin^1^ | Sulfa-trim^1^ | Trimeto-  prim^1^ | Tetra-  cycline^1^ |
| --- | --- | --- | --- | --- | --- | --- | --- | --- | --- | --- | --- | --- |
| Norway |  | | | | | | | | | | | |
| *S. aureus* | 20 | 1 | 0 | 0 | 0 | 0 | 1 | 0 | 1 | 0 | 1 | 0 |
| *S. chromogenes* | 2 | 0 | 0 | 0 | 0 | 1 | 0 | 0 | 1 | 0 | 0 | 0 |
| *S. epidermidis* | 2 | 0 | 0 | 0 | 0 | 0 | 0 | 0 | 1 | 1 | 1 | 0 |
| *S. simulans* | 2 | 0 | 0 | 1 | 0 | 0 | 0 | 0 | 0 | 1 | 2 | 0 |
| *S. warneri* | 2 | 0 | 0 | 0 | 0 | 0 | 1 | 0 | 1 | 0 | 0 | 3 |
| *S. hyicus* | 1 | 0 | 0 | 0 | 0 | 0 | 0 | 0 | 0 | 0 | 0 | 0 |
| *S. saprophyticus* | 1 | 0 | 0 | 0 | 0 | 0 | 0 | 0 | 1 | 0 | 0 | 0 |
| Belgium |  | | | | | | | | | | | |
| *S. aureus* | 25 | 3 | 3 | 4 | 4 | 4 | 2 | 0 | 5 | 12 | 17 | 1 |
| *S. haemolyticus* | 9 | 1 | 0 | 0 | 3 | 3 | 0 | 0 | 3 | 8 | 8 | 3 |
| *S. epidermidis* | 5 | 0 | 0 | 1 | 0 | 1 | 1 | 0 | 3 | 5 | 5 | 1 |
| *S. sciuri* | 5 | 0 | 0 | 0 | 1 | 0 | 2 | 0 | 3 | 5 | 5 | 4 |
| *S. chromogenes* | 4 | 0 | 0 | 0 | 1 | 1 | 0 | 0 | 2 | 2 | 2 | 1 |
| *S. hominis* | 4 | 0 | 0 | 1 | 0 | 1 | 1 | 0 | 0 | 4 | 4 | 2 |
| *S. cohnii* | 2 | 0 | 0 | 1 | 1 | 2 | 0 | 0 | 2 | 2 | 2 | 2 |
| *S. devriesei* | 2 | 0 | 0 | 0 | 0 | 0 | 0 | 0 | 1 | 1 | 2 | 0 |
| *S. equorum* | 2 | 1 | 0 | 0 | 1 | 2 | 0 | 0 | 0 | 2 | 2 | 2 |
| *S. warneri* | 2 | 0 | 0 | 0 | 2 | 2 | 0 | 0 | 2 | 0 | 0 | 2 |
| *S. xylosus* | 2 | 0 | 0 | 0 | 0 | 0 | 0 | 0 | 2 | 1 | 1 | 0 |
| *S. arlettae* | 1 | 0 | 0 | 0 | 1 | 1 | 0 | 0 | 1 | 0 | 0 | 0 |
| *S. auricularis* | 1 | 0 | 0 | 0 | 0 | 0 | 0 | 0 | 0 | 1 | 1 | 0 |
| *S. vitulinus* | 1 | 0 | 0 | 0 | 0 | 0 | 1 | 0 | 0 | 1 | 1 | 0 |
